# A Combined Controller for Closed-Loop Control Systems Affected by Electromagnetic Interference

**DOI:** 10.3390/s24051466

**Published:** 2024-02-24

**Authors:** Algirdas Baskys

**Affiliations:** 1 Faculty of Electronics, Vilnius Gediminas Technical University, Plytines Str. 25, 10105 Vilnius, Lithuania; algirdas.baskys@vilniustech.lt; 2 Department of Functional Materials and Electronics, Center for Physical Sciences and Technology, Sauletekio Ave. 3, 10257 Vilnius, Lithuania

**Keywords:** closed-loop control system, electromagnetic interference, noise signal, combined controller, noise signal resistance, robustness

## Abstract

In this paper, a new approach for the solution of the electromagnetic interference impact problem in closed-loop control systems with proportional-integral-derivative (PID) controllers is proposed. The approach is based on the application of a combined controller that consists of three controllers: PID, PI and I, when only one of them is operating at a time, and which one is operating determines the control error value. The proposed combined controller allows for achieving better resistance of the control system to the noise signals produced by electromagnetic interference compared to PID and PID with low-pass filters in derivative term controllers without deteriorating the dynamic performance of the control system. The operation of the controller has been analysed using simulation and experiments with plants, the dynamics of which are described by first-order plus dead-time transfer functions.

## 1. Introduction

Actual closed-loop control systems are often affected by electromagnetic interference, which produces a noise signal that sums with the input signal of the controller [1,2,3,4,5,6]. Often, this noise signal is called measurement noise. It can worsen the operation quality of the control system by causing ripples in the controlled parameter or even making the control system unstable. The problem of electromagnetic compatibility is particularly relevant in control systems used in power electronic devices that emit strong electromagnetic interferences. The proportional-integral-derivative (PID) controller, which is the most used in practice [7,8,9], is also widely used in power electronics [10,11,12,13,14]. It is well known that the derivative (D) term is the most sensitive to electromagnetic interference [15,16,17]. Therefore, this term is often excluded in practical applications, i.e., a PI controller is used instead of a PID controller [15,18,19]. However, excluding the derivative term worsens the dynamic performance of the control system; because of this, a low-pass filter is often used at the input of the derivative term to suppress noise signals. There are a lot of publications dedicated to the problem of noise signal filters for derivative terms, and first-order [20,21,22,23] or second-order [24,25] low-pass filters are usually used for this purpose. However, the low-pass filter worsens load disturbance rejection and lowers the robustness of the control system [25,26], and it does not always effectively suppress the ripples of the controlled parameter caused by the noise signal [27]. Also, the problem of electromagnetic compatibility in feedback control systems can be solved using shielding of the line through which the feedback signal from the sensor is supplied [28,29,30].

The contribution of the proposed approach is that the problem of electromagnetic compatibility of feedback control systems with PID controllers is proposed to be solved in a different way—by modifying the control algorithm so that it would not respond to noise signals and, at the same time, would not lose its control properties, i.e., that controller would properly respond to set point changes and load disturbances. The advantage of this approach is that improving the control system’s resistance to electromagnetic interference does not use classical EMI cancellation methods such as shielding or the application of low-pass filters, i.e., it does not require any material changes, it is enough only to modify the microcontroller program that implements the control algorithm.

The novelty of the work is that it proposes to use a combination of three controllers, PID, PI and I, instead of a single PID controller. Only one of these three controllers would be operating at a time, and which one is operating would determine the control error value. The proposed combined PID/PI/I controller allows for achieving better resistance of the control system to the noise signals produced by the electromagnetic interference compared to PID and PID with a low-pass filter in derivative term controllers without deteriorating the dynamic performance of the control system.

For the first time, the idea of applying combined controllers to solve the problem of electromagnetic compatibility of feedback control systems was presented by the author in the conference paper [31]. However, the conference paper proposes a different algorithm and analyses the effects of electromagnetic interference on a PI but not on a PID controller.

This paper is intended to show the electromagnetic compatibility capabilities of the proposed combined PID/PI/I controller and to demonstrate its advantages compared to the widely used PID controller using simulation and experiments.

## 2. Combined PID/PI/I Controller

**Figure 1:**
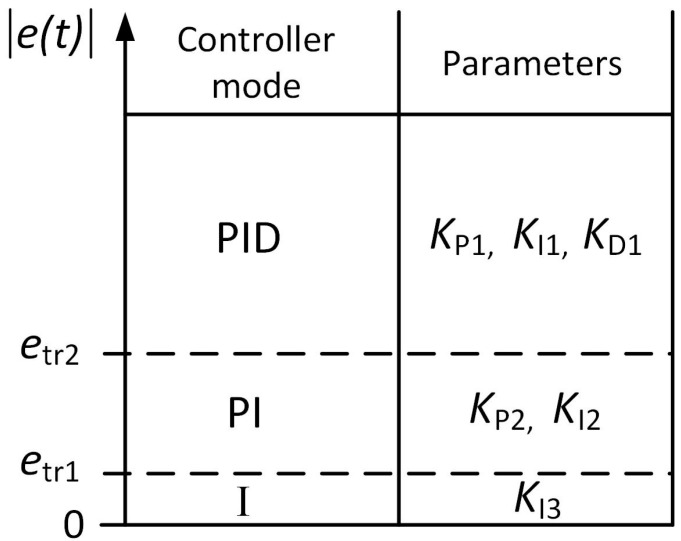
Modes of the combined PID/PI/I controller.

It is known that the I (integral) controller is more robust to noise signals compared to the PI (proportional-integral) controller, and the PI controller is more robust to noise signals than the PID controller, and, most of the time, the control system works in the state when the control error is low [31]. Since most of the working time, the controller operates in the mode when the control error is low, the I controller, which is most resistant to the noise signal, should be used in this mode. Since the I controller would be used only at low values of the control error, such a solution should not worsen the dynamic parameters of the control system. At slightly larger control error values, the PI controller could be used because it is sufficiently resistant to noise signals and, at the same time, guarantees sufficiently good dynamic parameters of the control system. In the remaining range of control error values, i.e., when the control error is large, a PID controller that guarantees the best control dynamics could be used. Since the control system works for a relatively short time in the mode when the control error is high (only during the starting of the control system and at the moment when a large load disturbance occurs), the PID controller would be applied only for a small part of the working time, i.e., the control system would be sensitive to noise signals for a relatively short period of the time. Based on the above considerations, a combined PID/PI/I controller was developed, and its properties were analysed using Matlab/Simulink software (version R2021b) and experimentally. The algorithm of the combined PID/PI/I controller is the following:

(1) $$U\left(t\right)=K_{P}\;e\left(t\right)+K_{I}\int_{t_{0}}^{t}e\left(t\right)\;dt+K_{D}\frac{de\left(t\right)}{dt},$$

$$K_{P}=K_{P1\;},\;{\;K}_{I}=K_{I1},\;{\;K}_{D}=K_{D1},\;\left|e\left(t\right)\right|\geq e_{tr2},$$

$$K_{P}=K_{P2\;},\;{\;K}_{I}=K_{I2},\;{\;K}_{D}=0,{\;e}_{tr2}>\left|e\left(t\right)\right|>e_{tr1},$$

$$K_{P}=0,\;{\;K}_{I}=K_{I3},\;{\;K}_{D}=0,\;\left|e\left(t\right)\right|\leq e_{tr1}$$

where *U*(*t*) is the controller output, *e*(*t*) is the control error (the difference between the set point and actual values of the parameter of the plant), *K*_P_, *K*_I_ and *K*_D_ are proportional, integral and derivative constants, respectively, *t* is time, *t*_0_ is the initial time moment, *e*_tr1_ and *e*_tr2_ are the threshold values of the control error *e*(*t*), at which the controller operation mode changes from I to PI and from PI to PID, or vice versa, respectively. Also, the condition *e*_tr1_ < *e*_tr2_ must be satisfied. For a better understanding of the operation of the combined PID/PI/I controller, the controller operation modes are presented graphically in Figure 1.

**Figure 2:**
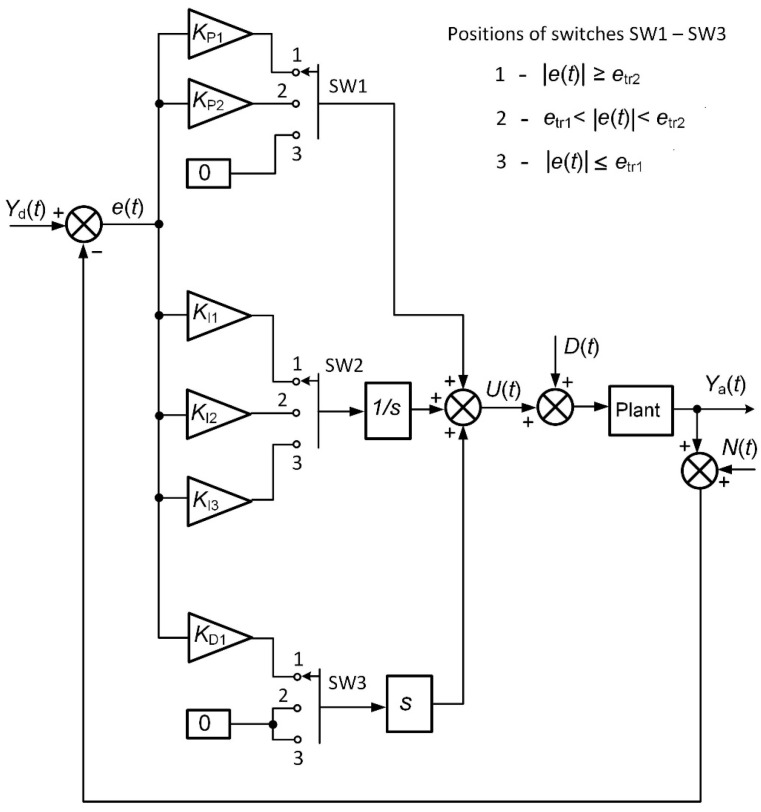
The block diagram of the control system with a combined PID/PI/I controller.

The block diagram of the closed-loop control system with a combined PID/PI/I controller is presented in Figure 2. There, in Figure 2, *Y*_d_(*t*) and *Y*_a_(*t*) are the set point (desired) and actual values of the controlled parameter of the plant, respectively; *D*(*t*) is the load disturbance, and *N*(*t*) is the noise signal induced by the electromagnetic interference. Usually, the noise signal is induced in the feedback circuit, i.e., in the line through which the feedback signal from the sensor measuring the controlled parameter of the plant is transmitted to the controller.

The controller modes are switched by switches SW1–SW3 (Figure 2). The position of the switches is determined by the value of the control error *e*(*t*). When |*e*(*t*)| ≥ *e*_tr2_, the switches SW1–SW3 are in position 1, so the combined controller works in PID controller mode with the parameters *K*_P_ *= K*_P1,_ *K*_I_ = *K*_I1_ and *K*_D_ = *K*_D1_. When *e*_tr2_ > |*e*(*t*)| > *e*_tr1_, the switches go to position 2, and the controller operates in PI controller mode with the parameters *K*_P_ *= K*_P2_ and *K*_I_ = *K*_I2_. At |*e*(*t*)| ≤ *e*_tr1_, the switches move to position 3, and the controller goes into I controller mode with the parameter *K*_I_ = *K*_I3_. The block diagram given in Figure 2 is presented in a way that would allow it to clearly reflect the essence of the proposed algorithm. When the control system is simulated using the Matlab/Simulink software, the switching function is performed with a multiport switch that measures the |*e*(*t*)| value and, when it reaches the *e*_tr1_ and *e*_tr2_ values, switches the constants of the controller according to control algorithm (1). In practice, the controller is developed using a microcontroller, which implements a control algorithm that provides the possibility to enter the required controller constants and error thresholds *e*_tr1_ and *e*_tr2_.

The work of the closed-loop control system based on the proposed combined PID/PI/I controller (Figure 2) was analysed with the plants, the dynamics of which are described by the first-order plus dead-time (FOPDT) transfer function. Such a model of the plant was chosen because it describes the dynamics of many real industrial processes [32,33,34,35,36,37]. The response delay (dead-time) values were chosen based on the literature [32,33,34,35,36,37], as well as from the practice of application of control systems. For example, the water supply systems for which this controller was developed had such response delay values, and their dynamics corresponded to the FOPDT transfer function. An investigation was performed with the three plants, described by the following FOPDT transfer functions with various dead times (response delays):

(2) $$G_{P1}\left(s\right)=\frac{e^{-1s}}{\left(s+1\right)}\;,\;$$

(3) $$G_{P2}\left(s\right)=\frac{e^{-3s}}{\left(s+1\right)}\;,\;$$

(4) $$G_{P3}\left(s\right)=\frac{e^{-5s}}{\left(s+1\right)}\;.\;$$

**Figure 3:**
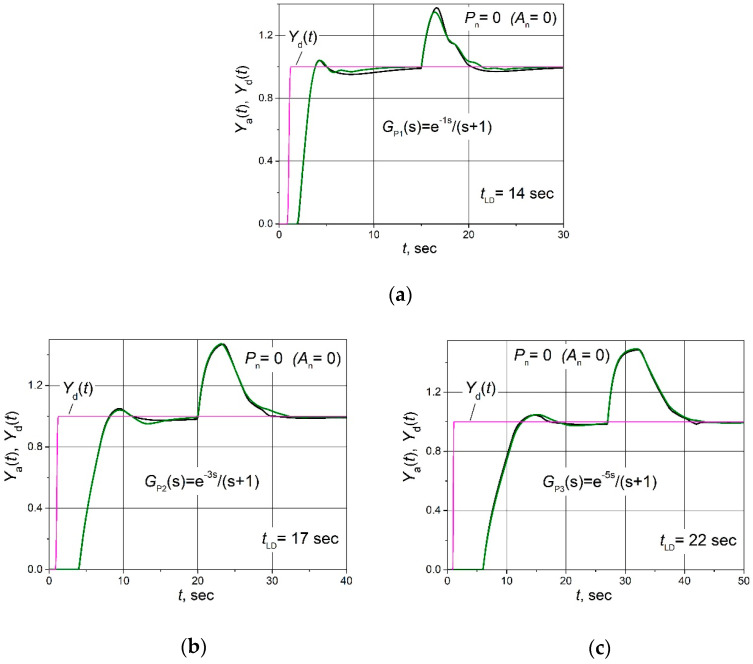
The set point unit step (purple lines) response followed by the 0.5 load step disturbance of the control systems of plants *G*_P1_(s) (**a**), *G*_P2_(s) (**b**) and *G*_P3_(s) (**c**) based on the combined PID/PI/I (black lines) and PID (green lines) controllers, when the control system is not affected by the electromagnetic interference (amplitude of noise signal *A*_n_ = 0).

**Figure 4:**
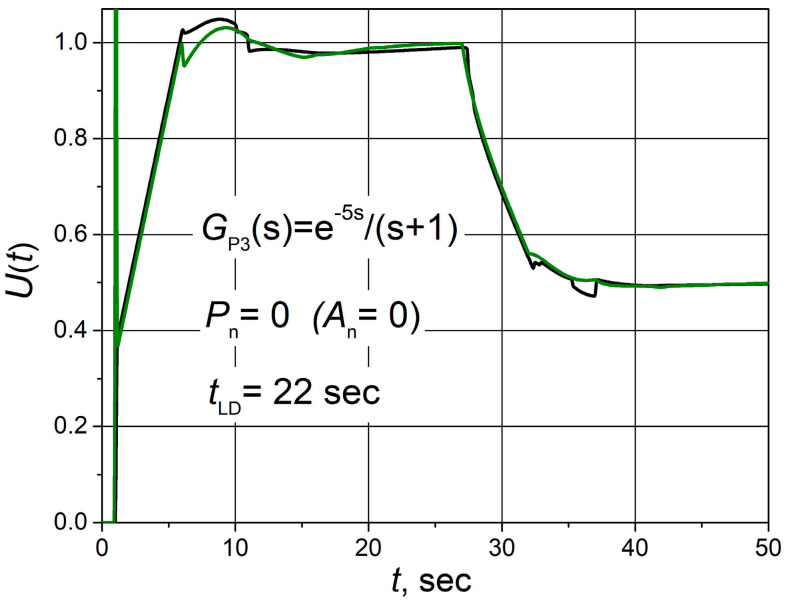
Output signals of PID/PI/I (black) and PID (green) controllers during the set point unit step response followed by the 0.5 load step disturbance of the control system with the plant *G*_P3_(s).

First of all, the dynamic performance of the control system was analysed when the system was not affected by electromagnetic interference (noise signal power *P*_n_ = 0). For that purpose, the set point *Y*_d_(*t*) unit step response, followed by the 0.5 load step disturbance *D*(*t*) for the control system with the plants described by transfer Functions (2)–(4), was obtained when combined PID/PI/I and PID controllers are used. The obtained transients are presented in Figure 3, where *t*_LD_ is the moment in time when the load disturbance occurs. The transients of output signals *U*(*t*) of PID/PI/I and PID controllers for the control system with the plant *G*_P3_(s) are presented in Figure 4. The parameters of the controllers are given in Table 1.

From the obtained results, it is seen (Figure 3) that the response transients of the control system using the combined PID/PI/I controller and the PID controller practically coincide. Therefore, it can be stated that the dynamic parameters of the control system with the analysed plants using the combined PID/PI/I controller are the same as when using the PID controller, i.e., excluding the D term, and then, the P term at a low value of the control error *e*(*t*) does not cause a noticeable deterioration in the dynamics of the control system.

The transients of output signals of PID/PI/I and PID controllers *U*(*t*) coincide in the initial part of the transient (Figure 4) because at the beginning of the response, when the value of the control error *e*(*t*) is high (*e*(*t*)> *e*_tr2_), the PID/PI/I controller operates in PID mode. However, as *e*(*t*) decreases later, the PID/PI/I controller switches to PI and then to I modes, so the responses of *U*(*t*) no longer match.

The parameters (gains) of the combined PID/PI/I and PID controller were tuned to obtain the minimal set point step response settling time when the overshoot does not exceed 5%. Analysis has shown that PID/PI/I controller parameters *K*_P1_, *K*_I1_ and *K*_D1_, which are used when the controller operates in a PID mode, coincide with PID controller parameters *K*_P_, *K*_I_ and *K*_D_ (see Table 1). Therefore, the methods of the PID controller tuning can be used to determine the values of these parameters. The remaining parameters *K*_P2_, *K*_I2_ and *K*_I3_, which are used when the system works in PI and I modes, respectively, have less influence on the dynamics of the control system because in those modes, the controller works at low control errors. Analysing the studied control systems with a PID/PI/I controller, it was found that when tuning the controller for a minimum set point response settling time, the parameter *K*_I2_ should be about 40% higher than *K*_I1_, and *K*_P2_ should be about 30% lower than *K*_P1_. Parameter *K*_I3_ determines the resistance of the control system to the influence of noise signals when the control system operates at low control error, i.e., in the steady state mode. Since *K*_I3_ has little influence on the dynamics of the system, it is appropriate to choose a significantly reduced value to lower the sensitivity of the controller to the noise signals. In this work, *K*_I3_ was chosen two to three times lower compared to *K*_I1_.

Studies have shown that the threshold values of the control error *e*_tr1_ and *e*_tr2_, at which the controller operation mode changes from I to PI and from PI to PID, or vice versa, respectively, should be selected so that *e*_tr2_ would be close to the amplitude of noise signals; then the control system would not respond to noise signals while working in the steady state mode. Meanwhile, the value of *e*_tr1_ was chosen to be about two times lower than that of *e*_tr2_.

## 3. Analysis of Resistance to Electromagnetic Interference of the Control System Based on the Combined PID/PI/I Controller

**Figure 5:**
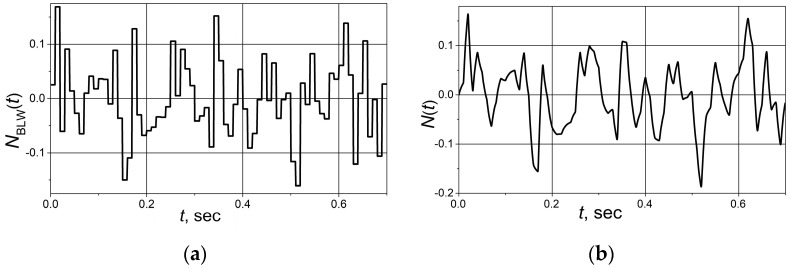
The band-limited white noise with seed [23341], sample time 10^−2^ s. (**a**) and filtered band-limited white noise signal using low-pass filter with transfer function *G*_f_(s) = 1.5/(0.01 s + 1) (**b**).

The operation of the control system based on the combined PID/PI/I controller was investigated when the control system was affected by the noise signal *N*(*t*) induced by electromagnetic interference. The analysis was carried out with the FOPDT plants, the dynamics of which are described by transfer Functions (2)–(4). The results were obtained for the band-limited white noise signal, which adds up with the feedback signal of the control system (Figure 2). To make the noise signal similar to the actual one, the band-limited white noise signal was filtered using a first-order low-pass filter, as suggested in the reference [27]. The curves of the band-limited white noise signal and filtered noise signal are presented in Figure 5. The results were obtained for the cases when the noise signal begins to act after the set point step response transient process of the control system is over. The obtained results were compared with those gained using PID controller. The parameters of the controllers are given in Table 1.

**Figure 6:**
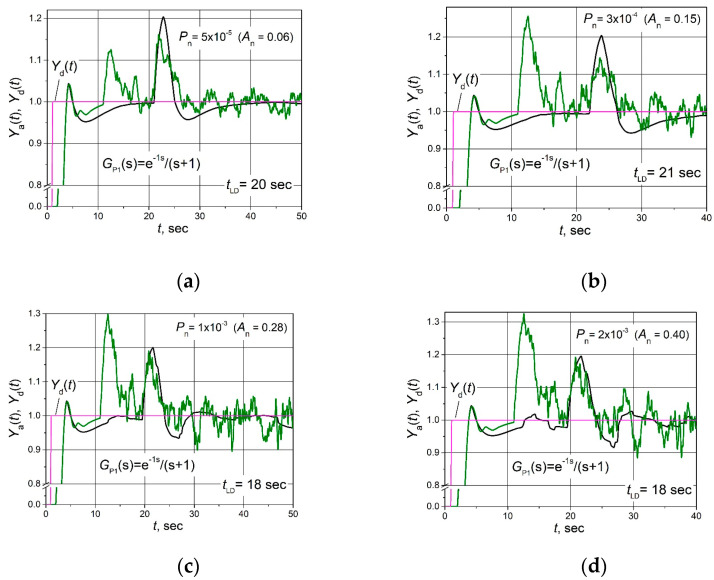
The set point unit step (purple lines) response followed by the 0.25 load step disturbance of the control system of the plant *G*_P1_(s) based on the combined PID/PI/I (black lines) and PID (green lines) controllers when the control system is affected by the noise signal with amplitudes *A*_n_ = 0.06 (**a**), 0.15 (**b**), 0.28 (**c**) and 0.40 (**d**).

**Figure 7:**
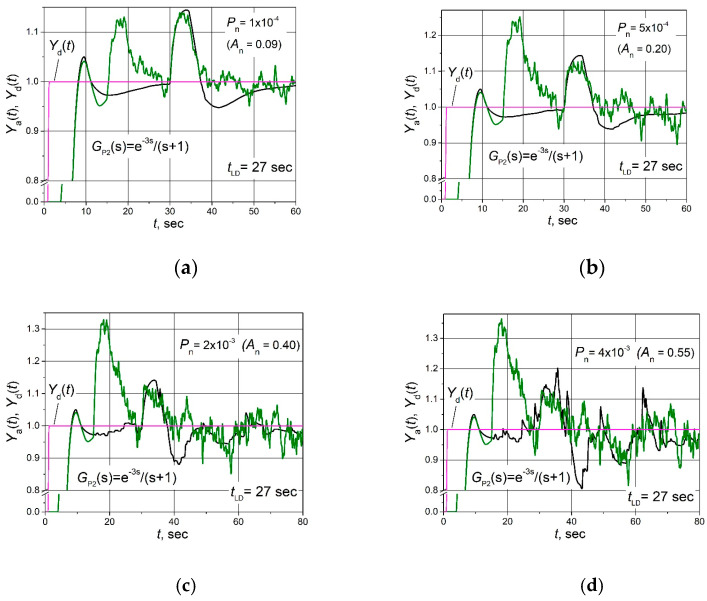
The set point unit step (purple lines) response followed by the 0.15 load step disturbance of the control system of plant *G*_P2_(s) based on the combined PID/PI/I (black lines) and PID (green lines) controllers when the control system is affected by the noise signal with amplitudes *A*_n_ = 0.09 (**a**), 0.20 (**b**), 0.40 (**c**) and 0.55 (**d**).

**Figure 8:**
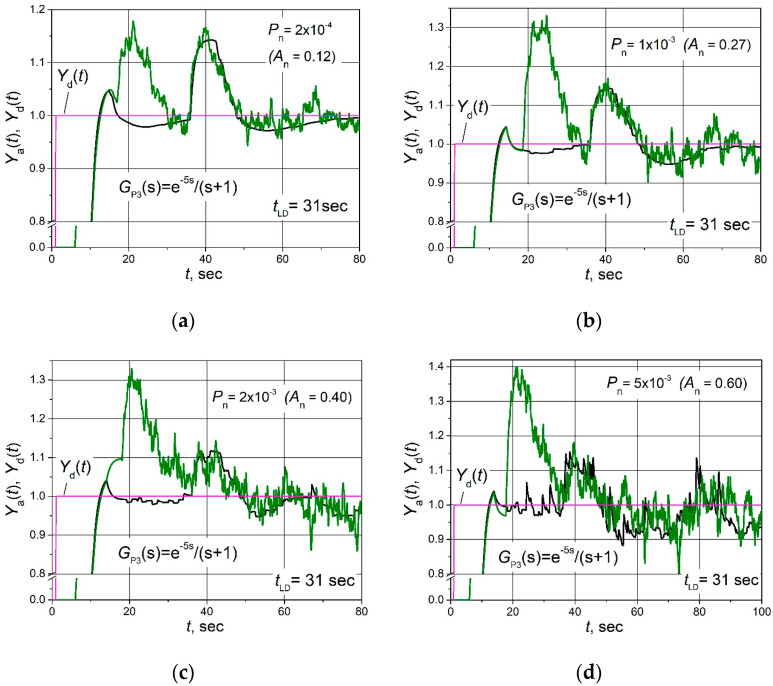
The set point unit step (purple lines) response followed by the 0.15 load step disturbance of the control system of plant *G*_P3_(s) based on the combined PID/PI/I (black lines) and PID (green lines) controllers, when the control system is affected by the noise signal with amplitudes *A*_n_ = 0.12 (**a**), 0.27 (**b**), 0.40 (**c**) and 0.60 (**d**).

The set point *Y*_d_(*t*) unit step responses, followed by the 0.25 load step disturbance *D*(*t*) response for the control systems with FOPDT plants described by the transfer Functions (2)–(4) based on the combined PID/PI/I and PID controllers affected by the filtered band-limited white noise signal *N*(*t*) (Figure 5b), are given in Figure 6, Figure 7 and Figure 8.

It is seen (Figure 6b, Figure 7b and Figure 8b) that in the cases when the amplitude of the noise signal *A*_n_ ≤ (0.15–0.27), the noise signal does not cause any ripples of the plant parameter *Y*_a_(*t*) if the combined PID/PI/I controller is used. On the other hand, when a PID controller is applied at the same noise signal values, the plant parameter *Y*_a_(*t*) pulsates, and the maximal amplitude of ripples reaches values from 0.25 to 0.32 (Figure 6b, Figure 7b and Figure 8b). When the noise signal amplitude increases up to *A*_n_ = (0.28–0.40), ripples of the plant parameter *Y*_a_(*t*) also appear in the system with the combined PID/PI/I controller, and the amplitude of the ripples is in the range 0.06 to 0.11, depending on the plant. However, these values are significantly lower than using the PID controller when amplitude reaches a value of 0.3 for control systems with all analysed plants (Figure 6c, Figure 7c and Figure 8c). In the case when noise signal amplitudes rise up to values *A*_n_ = (0.40–0.60), the ripple amplitudes of the plant parameter *Y*_a_(t) using the combined PID/PI/I controller become close to those obtained using the PID controller, except for the first overshoot, which reaches values from 0.32 to 0.4 when the PID controller is used (Figure 6d, Figure 7d and Figure 8d).

Summarizing the obtained results, it can be stated that the proposed combined PID/PI/I controller provides the same dynamic properties of the analysed control system as the PID controller but guarantees higher resistance to noise signals when the amplitude of the noise signal *A*_n_ < (0.28–0.40).

**Figure 9:**
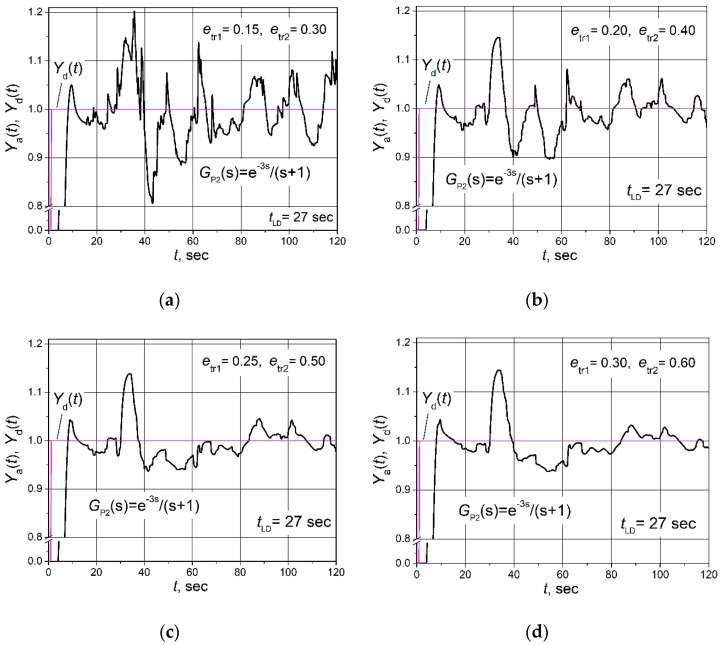
The set point unit step (purple lines) response followed by the 0.15 load step disturbance of the control system of the plant *G*_P2_(s) based on the combined PID/PI/I controller (black lines) at values of controller parameters *e*_tr1_ = 0.15, *e*_tr2_ = 0.30 (**a**), *e*_tr1_ = 0.20, *e*_tr2_ = 0.40 (**b**), *e*_tr1_ = 0.25, *e*_tr2_ = 0.50 (**c**) and *e*_tr1_ = 0.30, *e*_tr2_ = 0.60 (**d**), when the control system is affected by the noise signal with power *P*_n_ = 0.004 (amplitude *A*_n_ = 0.55).

The amplitude of the noise signal at which ripples of the plant parameter *Y*_a_(t) appear should depend on the values of the parameters *e*_tr1_ and *e*_tr2_, at which the combined PID/PI/I controller switches from I mode to PI mode and from PI mode to a PID mode. This should happen because the control error *e*(*t*) = *Y*_d_(t) − *Y*_a_(t) − *N*(t) (see Figure 2). If the control system is in the steady state, i.e., *Y*_a_(t)≈*Y*_d_(t), and a noise signal appears, the initial instantaneous value of control error *e*(*t*)≈*N*(t). If *N*(t) > *e*_tr1_, according to (1) and Figure 1, the combined PID/PI/I controller switches from I mode to PI mode; therefore, it becomes more sensitive to the noise signal, and if *N*(t) > *e*_tr2_ it becomes even more sensitive because it switches to PID mode. Because of this, the noise signal resistance of the control system based on the combined PID/PI/I controller should increase with increasing values of *e*_tr1_ and *e*_tr2_. This hypothesis was tested by investigating the influence of the noise signal on the ripples of the plant parameter *Y*_a_(t) at various values of parameters *e*_tr1_ and *e*_tr2_. The values of other parameters of the controller are given in Table 1. The results are presented in Figure 9.

The obtained results show that the ripples of the plant parameter *Y*_a_(t) decrease with increasing *e*_tr1_ and *e*_tr2_ values. The amplitude of ripples reaches 0.2 when *e*_tr1_ = 0.15 and *e*_tr2_ = 0.30 (Figure 9a), and when they are increased to *e*_tr1_ = 0.25 and *e*_tr2_ = 0.50, the maximal amplitude of ripples decreased to 0.05 (Figure 9c). It can also be seen that after the increasing of values from *e*_tr1_ = 0.25 and *e*_tr2_ = 0.50 to *e*_tr1_ = 0.30 and *e*_tr2_ = 0.60, the amplitude of *Y*_a_(t) ripples practically did not decrease (compare the dependence presented in Figure 9c with this one given in Figure 9d).

When choosing the parameters *e*_tr1_ and *e*_tr2_ of the combined PID/PI/I controller, it is necessary to consider that increasing their values worsens the dynamic properties of the control system during the set point change and load disturbance response. This occurs because the controller switches from PID mode to PI and I modes at higher control error *e*(*t*) values during the response, i.e., the proportion of the time when the controller works in PI and I controller modes increases compared to the proportion when it works in PID mode. Therefore, it is necessary to choose the values of the parameters *e*_tr1_ and *e*_tr2_ based on a compromise between the resistance to the noise signal and the dynamic properties of the control system.

As was mentioned in the introduction of the article, a first-order or second-order low-pass filter is often used at the input of the D (derivative) term to increase the resistance of the control system with a PID controller to noise signals. The analysis was conducted in order to compare the resistance to noise signals of the control system with the proposed combined PID/PI/I controller with the noise resistance when the control system is based on the PID controller with a low-pass filter in the D term. The analysis was performed using a first-order low-pass filter for control systems with all analysed FOPDT plants described by the transfer Functions (2)–(4). The transfer function of the filter was calculated according to the formula [20,38] *G*_F_(*s*) = 1/[(0.1 *s K*_d_/*K*_p_) + 1]. The transfer functions of the filters used in derivative terms of the PID controller were as follows: 1/(0.104 s + 1), 1/(0.14 s + 1) and 1/(0.114 s + 1) for control systems of plants *G*_P1_(s), *G*_P2_(s) and *G*_P3_(s), respectively.

**Figure 10:**
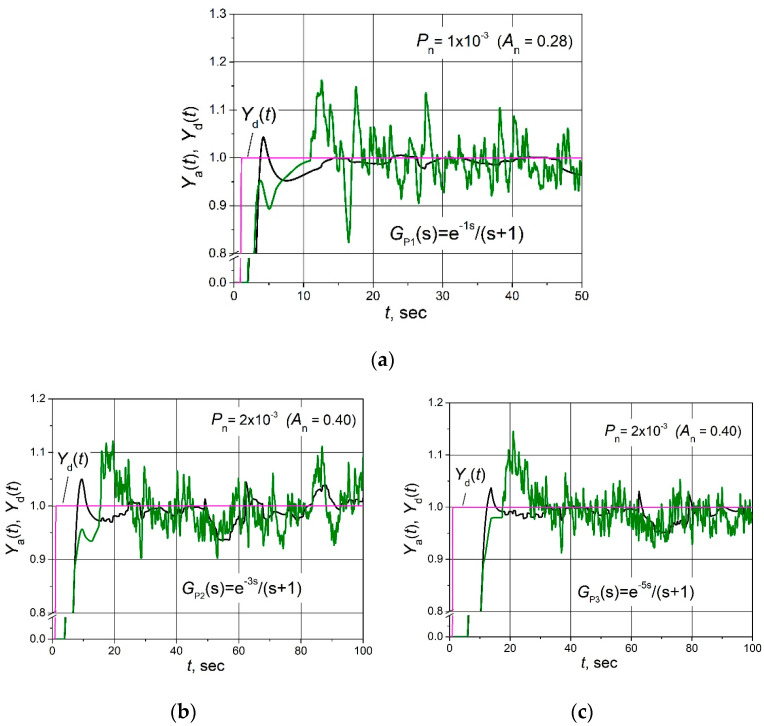
The set point unit step (purple lines) response of the control system with plants *G*_P1_(s) (**a**), *G*_P2_(s) (**b**) and *G*_P3_(s) (**c**) based on the combined PID/PI/I controller (black lines) and PID controller with a low-pass filter in the derivative term (green lines) affected by the noise signal with the amplitude *A*_n_.

The obtained results are presented in Figure 10. The amplitudes of the noise signal at which the tests were performed were chosen to be relatively high (*A*_n_ = 0.28 and 0.40) in order to observe ripples using both tested controllers.

From the obtained results, we can see that the application of a low-pass filter in the D term reduces slightly the maximum amplitude of ripples of the plant parameter *Y*_a_(*t*) of the control system with a PID controller (compare the curves for PID controller presented in Figure 10a–c with those given in Figure 6c, Figure 7c and Figure 8c, respectively). However, the application of the proposed combined PID/PI/I controller guarantees lower ripples of *Y*_a_(*t*). We can see (Figure 10) that the maximum amplitudes of ripples using the PID/PI/I controller are two to four times lower compared to the case when the PID controller with a low-pass filter for the D term is used.

## 4. Investigation of Robustness of Control Systems Based on the Combined PID/PI/I Controller

Robustness is a very important parameter of the control system. It characterizes the ability of the control system to operate stably when plant parameters change. It makes sense to investigate the response of the control system in the time domain when dynamic parameters of the controlled plant change and determine boundaries within which the stability of the control system is guaranteed. During the set point change response, when the control error value changes, the controller goes through all working modes (PID, PI and I), as well as through transitions when modes are switched. Since such an analysis is carried out in the way in which the real control system works, the obtained results have to be reliable.

The robustness of the control system with a combined PID/PI/I controller was studied when both the plant response delay and plant time constant change. The most dangerous for the operation stability of the control system is the increase in plant response delay. Since the parameters of the controller do not change, the controller becomes too aggressive in such a case. Therefore, the transient duration of the control system response may increase, and the amplitude of *Y*_a_(*t*) oscillations may rise during it. Also, the system may become unstable if the plant response delay increases significantly.

**Figure 11:**
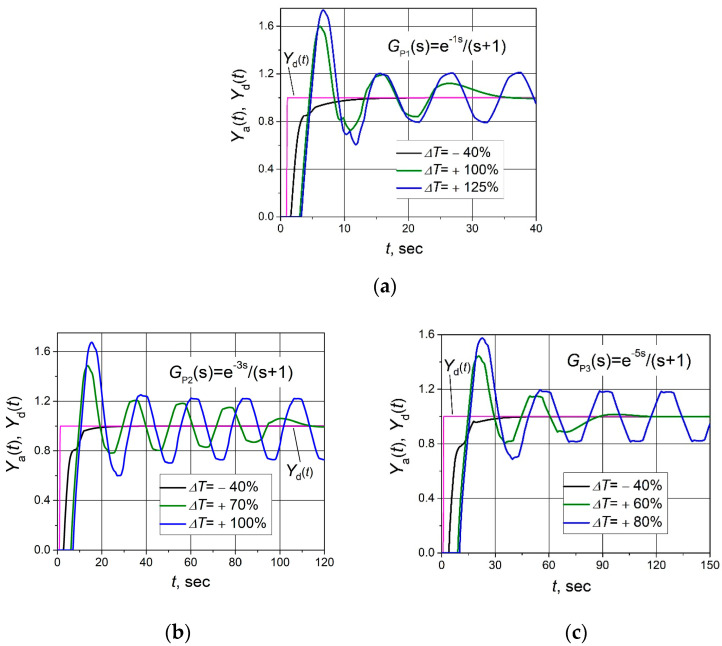
The set point unit step (purple lines) response of the control system with plants *G*_P1_(s) (**a**), *G*_P2_(s) (**b**) and *G*_P3_(s) (**c**) based on the combined PID/PI/I controller when plant response delay changes by ΔT value.

The investigation results of the control system with a combined PID/PI/I controller when the plant response delay changes and controller parameters remain unchanged (parameters of the controller are given in Table 1) are presented in Figure 11. It is seen that when the plant response delay increases, the duration of the transient process of the control system’s response and the amplitude of the oscillations increase.

The control system with plants *G*_P1_(s), *G*_P2_(s) and *G*_P3_(s) remains stable when the increase in response delay of the plant does not exceed 100%, 70% and 60%, respectively (Figure 11). When the increase in response delay of plants *G*_P1_(s), *G*_P2_(s) and *G*_P3_(s) reaches 125%, 100% and 80%, respectively, the control systems of these plants start to operate unstably.

**Figure 12:**
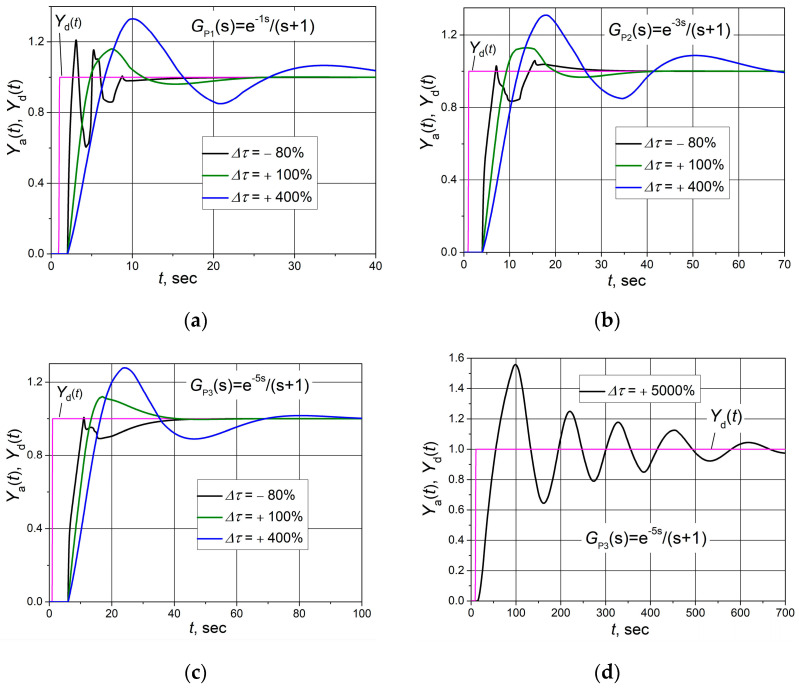
The set point unit step (purple lines) response of the control system with plants *G*_P1_(s) (**a**), *G*_P2_(s) (**b**) and *G*_P3_(s) (**c**,**d**) based on the combined PID/PI/I controller when plant time constant changes by Δτ value.

The operation stability of the control system is also influenced by the variation in the plant time constant. Therefore, analyses of the control system with a combined PID/PI/I controller were carried out when the plant time constant changed. The obtained results are presented in Figure 12. We can see that the variation in the plant time constant has a weak influence on the stability of the control system’s work with all analysed plants. For example, the control system with plant *G*_P3_ (Figure 12d) remains stable even when the time constant increases 50 times. As the time constant increases, only the overshoot and transient process duration increase, and the oscillation period also increases.

**Figure 13:**
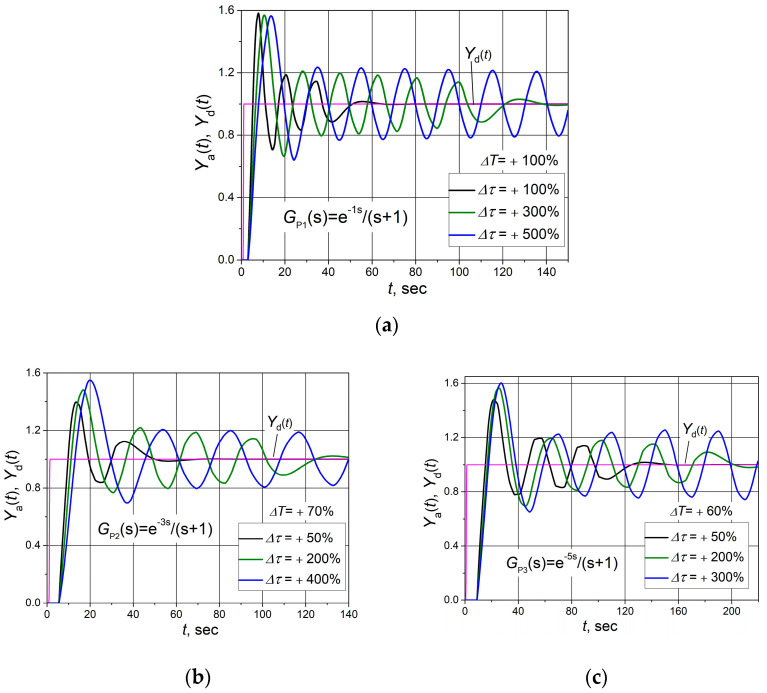
The set point unit step (purple lines) response of the control system with plants *G*_P1_(s) (**a**), *G*_P2_(s) (**b**) and *G*_P3_(s) (**c**) based on the combined PID/PI/I controller when the plant response delay increases by the value Δ*T* and plant time constant rises by Δ*τ* value.

In practice, both the plant response delay and the plant time constant may change when the plant operating conditions change. Therefore, studies of the control system were carried out when both of these plant dynamic parameters varied. Cases were analysed when the plant response delay increases to a value close to the limit at which the control system becomes unstable, and then the time constant of the plant is increased. This study aimed to find the limit values of the plant time constant at which the system becomes unstable in this situation. The values of the change in time constants Δ*T* at which the tests were carried out are taken from the results presented in Figure 11. It is seen (Figure 13) that with a significant increase in the plant response delay time (by 60–100%), the control system may become unstable with an increase in the plant time constant. In the studied cases, depending on the analysed plant, the control system became unstable when the time constant increased (by 300–500%).

Based on the obtained results (Figure 11 and Figure 13), it can be stated that control systems of plants *G*_P1_, *G*_P2_ and *G*_P3_ based on the combined PID/PI/I controller will work stably if the dynamic parameters of the plants are bounded by values Δ*T* < +100%, Δτ < +300%; Δ*T* < +70%, Δτ < +200% and Δ*T* < +60%, Δτ < +200%, respectively.

## 5. Frequency Response Analysis

**Figure 14:**
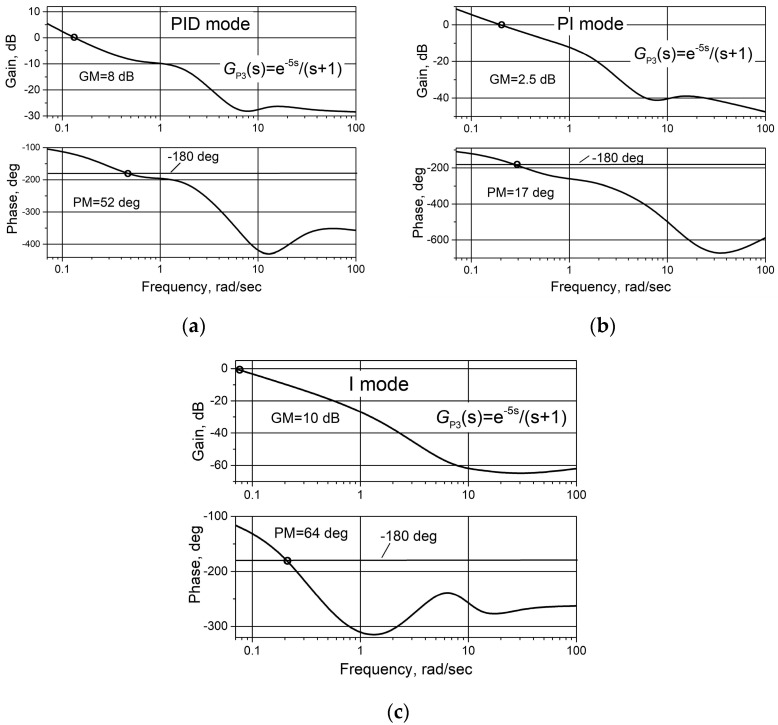
Open-loop Bode diagrams of the control system with plant *G*_P3_(s) based on the combined PID/PI/I controller when the controller operates in PID (**a**), PI (**b**) and I (**c**) modes. GM is the gain margin; PM is the phase margin.

The frequency response of the control system with PID/PI/I controller was studied. The analysis was provided separately for each operating mode of the controller, i.e., for PID, PI and I modes. The frequency responses of the control system with plant *G*_P3_(s) were obtained because the results presented in the previous section of the article show that the control system with the plant *G*_P3_(s) is characterized by the lowest robustness. The obtained open-loop Bode diagrams are presented in Figure 14. It is seen that the control system has the highest gain (GM) and phase (PM) margins when operating in I mode (GM = 10 dB, PM = 64 deg) and the lowest in PI mode (GM = 2.5 dB, PM = 17 deg).

**Figure 15:**
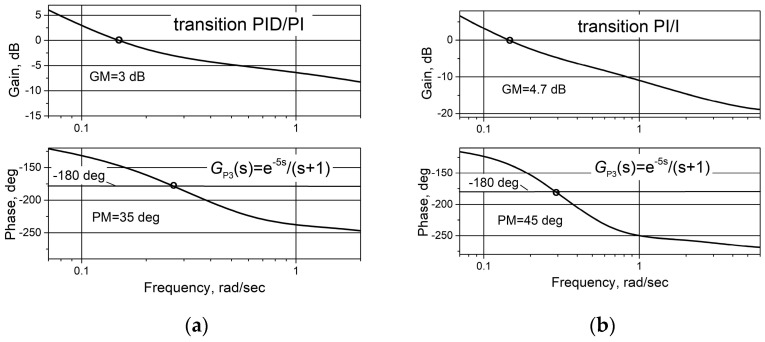
Open-loop Bode diagrams of the control system with plant G_P3_(s) based on the combined PID/PI/I controller when the controller is in transition between PID and PI (**a**), PI and I (**b**) modes.

At the time moments when the controller switches between PID and PI and between PI and I operating modes, the control system becomes nonlinear. However, analysis of frequency responses shows that during the transition between PID and PI, the sine shape of the response signal is almost undistorted when the frequency is not higher than 2 rad/s and during the transition between PI and I—if it is not higher than 6 rad/s. This means that the frequency response method is applicable at the mentioned frequencies. The open-loop Bode diagrams when the PID/PI/I controller is in transition between operating modes are shown in Figure 15. It can be seen that the gain crosses the zero value and the phase—the value of −180 deg at a frequency lower than 0.2 rad/s, so the gain (GM) and phase (PM) margins can be determined from the obtained Bode diagrams. It is seen that GM and PM values are higher during the transition between PI and I as compared to the values obtained for the transition between PID and PI.

**Figure 16:**
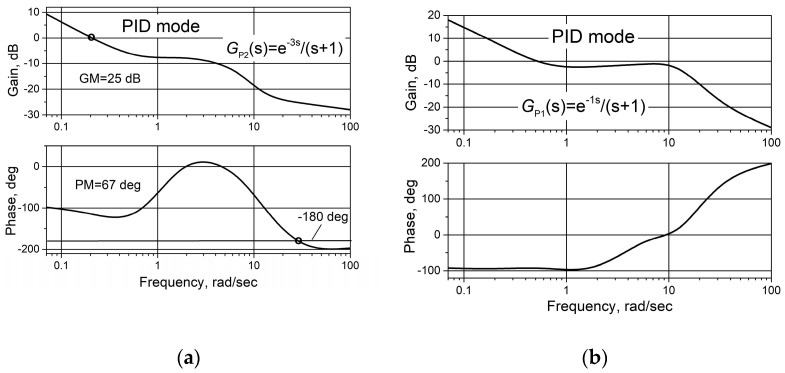
Open-loop Bode diagrams of the control system with plants G_P1_(s) (**a**) and G_P2_(s) (**b**) based on the combined PID/PI/I controller when the controller operates in a PID mode.

Figure 16 shows the open-loop Bode diagrams for control systems with plants G_P1_(s) (a) and G_P2_(s) when the PID/PI/I controller is operating in PID mode. Since the analysed PID controller has the same values of parameters as the PID/PI/I controller in a PID mode (Table 1), the diagrams presented in Figure 14a and Figure 16 also are valid for the PID controller.

## 6. Experimental Verification

The performance of the combined PID/PI/I controller was verified using it in a frequency converter for AC induction motor speed control. The AC motor is used in the pump drive of the water supply system. The controller has to control the speed of the pump in a feedback control system using a frequency converter to maintain a preset (desired) value of water pressure. The water pressure was measured using a transducer with a 0–10 bar measurement range and a 0–10 V analogue output (1 bar of water pressure corresponds to 1 V of the sensor output signal). The response of the water pressure control system (signal of pressure transducer) was measured using a USB PC-oscilloscope PicoScope 2206b. A noise signal *N*(*t*) similar to the one shown in Figure 5b was generated using the function generator TDS1012B. Water pressure set point step responses were obtained when the noise signal was summed with the controller feedback signal sent from the water pressure sensor.

**Figure 17:**
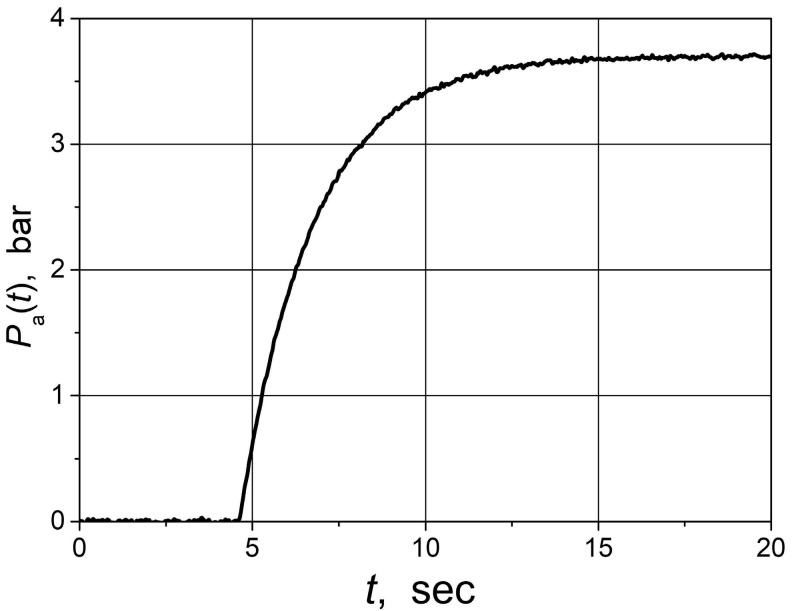
Step response of the water supply system.

The water pressure (*P*_a_(t)) step response of the water supply system is presented in Figure 17. The *P*_a_(*t*) responds with the 4.5 s delay. The dynamics of the water supply system corresponds with the first-order transfer function *G*_PW_ = e^−4.5^/(2 s + 1).

**Figure 18:**
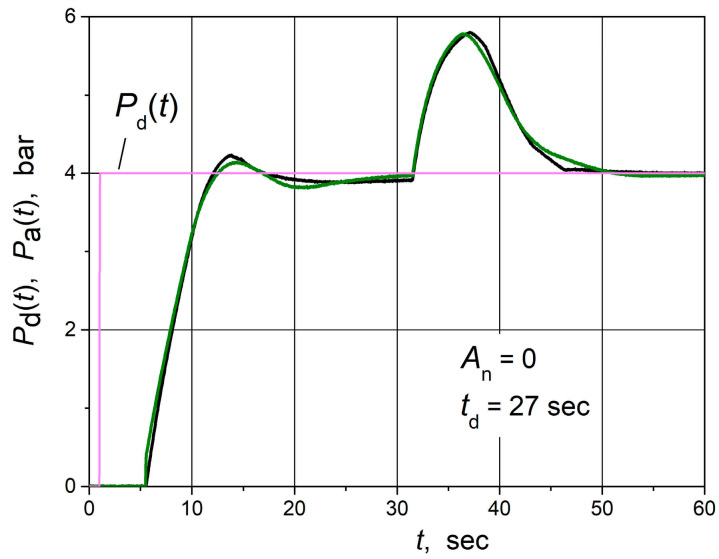
The water pressure set point step (purple line) response followed by the load step disturbance of the water supply control system based on the combined PID/PI/I (black line) and PID (green line) controllers when the control system is not affected by the noise signal (*A*_n_ = 0). *P*_d_(*t*) is desired (set point), and *P*_a_(*t*) is the actual value of water pressure, respectively.

The water pressure set point (*P*_d_(*t*)) step responses followed by the load step disturbance of the water supply control system based on the combined PID/PI/I and PID controllers are given in Figure 18. The load disturbance was introduced by closing one of the valves in the water supply system. The parameters of the controllers are presented in Table 2. It is seen that responses of the control system using both controllers practically coincide, i.e., the application of the combined PID/PI/I controller instead of the PID controller does not worsen the dynamic performance of the analysed control system.

**Figure 19:**
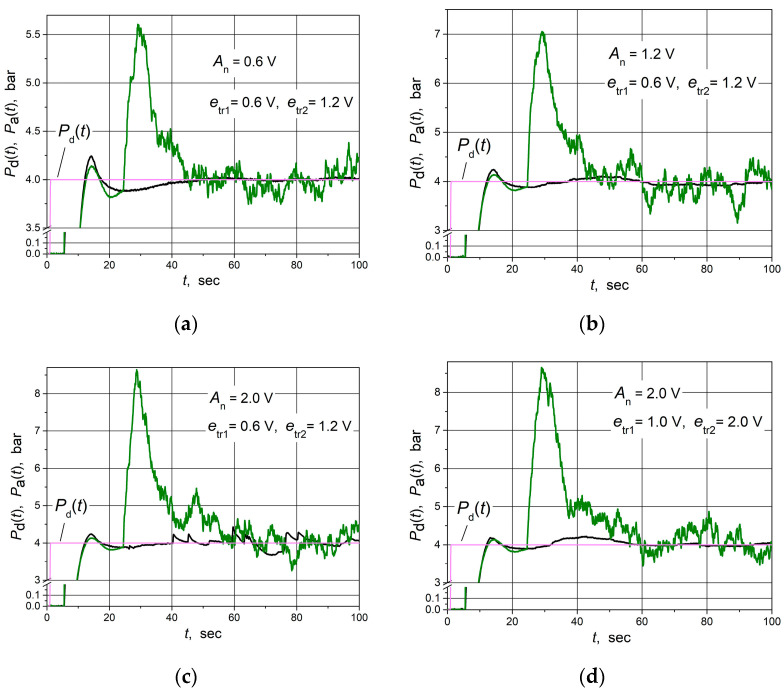
The set point step (purple lines) response of the water supply control system based on the combined PID/PI/I controller (black lines) and PID controller (green lines) affected by the noise signal with amplitudes *A*_n_ = 0.6 V (**a**), 1.2 V (**b**) and 2.0 V (**c**,**d**).

The responses of the water control system based on the combined PID/PI/I controller at various noise signal amplitudes are presented in Figure 19. They are compared with the responses when the PID controller is used. The obtained experimental results support the simulation results. It is seen that the water supply control system based on the proposed combined PID/PI/I controller is more robust to noise signals than the control system with the PID controller. The response of the control system with the PID/PI/I controller presented in Figure 19d is obtained at the same noise signal amplitude *A*_n_ = 2 V as in Figure 19c but at higher values of the control error thresholds *e*_tr1_ and *e*_tr2_. It can be seen that when the *e*_tr1_ and *e*_tr2_ values are increased, the water pressure ripples disappear (compare the transients shown in black in Figure 19c,d). These results confirm the results obtained using simulation. It is necessary to note that the parameters of controllers for which Figure 19 transients are obtained are presented in Table 2, except for the Figure 19d transient (black) that is obtained for the increased *e*_tr1_ and *e*_tr2_ values, which are indicated in the figure.

## 7. Discussion

Actual closed-loop control systems, which are mostly based on the PID controllers, are often affected by electromagnetic interference that introduces the noise signals on the input of the controller. However, not much attention is paid to the problem of electromagnetic interference in works on control systems. Most often, the existence of this problem and the importance of its solution become known when working with industrial control systems, especially when closed-loop control systems are applied in power electronics devices. A popular solution that has been applied in practice and is the subject of publications is the filtering of a noise signal using a low-pass filter for D (derivative) term, which is the most sensitive to noise signals. However, application of the low-pass filter worsens load disturbance rejection and robustness of the control system and it not always effectively suppresses the ripples of the plant parameter *Y*_a_(*t*) caused by the noise signal.

A new approach for solving the electromagnetic interference problem in closed-loop control systems is used in this work. It is based on the use of the control algorithm that provides an adequate response to set point and load disturbance signals and responds weakly to noise signals. The proposed algorithm uses a combination of PID, PI and I controllers: the I controller is used when the control error *e*(*t*) is low, i.e., the control system works in the steady state mode; PI is used at slightly larger *e*(*t*) values; PID is applied when *e*(*t*) is large. Simulation and experimental investigation results show that dynamic properties of the control system with the analysed FOPDT plants using the combined PID/PI/I controller are the same as when using the PID controller, i.e., on the one hand, excluding the D term and then the P term at low values of the *e*(*t*) does not cause a noticeable deterioration of the dynamics of the control system, and, on the other hand, it allows increasing of the resistance of the control system to electromagnetic interference when the control system operates in a steady-state mode or close to it.

The obtained results demonstrate that in the case when a combined PID/PI/I controller is used for the control of analysed FOPDT plants, the noise signal, which adds up to the feedback signal, does not cause any ripples of the plant parameter *Y*_a_(*t*) when the amplitude of the noise signal *A_n_* ≤ (0.15–0.27). However, when a PID controller is applied at the same noise signal amplitude values, the plant parameter *Y*_a_(*t*) pulsates, and the maximal amplitude of ripples reaches a value of 0.25. A comparison of a combined PID/PI/I controller with a PID having a low-pass filter in the D term shows that the amplitudes of *Y*_a_(*t*) ripples caused by the noise signal for the control system with analysed plants using the PID/PI/I controller are two to four times lower compared to the case when the PID controller with a filter is applied.

The analysis of the robustness of control systems with analysed FOPDT plants *G*_P1_, *G*_P2_ and *G*_P3_ based on the combined PID/PI/I controller shows that systems will work stably if the dynamic parameters of the plants are bounded by values Δ*T* < +100%, Δτ < +300%; Δ*T* < +70%, Δτ < +200% and Δ*T* < +60%, Δτ < +200%, respectively.

Since the problem of electromagnetic compatibility is often encountered in the application of closed-loop control systems, and there are not many publications devoted to this problem, it is likely that the material presented in the article may be of interest to a wider audience.

## Figures and Tables

**Table 1:** Parameters of controllers.

	Plant	*G*_P1_(*s*)	*G*_P2_(*s*)	*G*_P3_(*s*)
Controller				
PID/PI/I		*K*_P1_ = 0.77	*K*_P1_ = 0.44	*K*_P1_ = 0.35
		*K*_I1_ = 0.54	*K*_I1_ = 0.207	*K*_I1_ = 0.132
		*K*_D1_ = 0.80	*K*_D1_ = 0.50	*K*_D1_ = 0.40
		*K*_P2_ = 0.40	*K*_P2_ = 0.30	*K*_P2_ = 0.30
		*K*_I2_ = 0.70	*K*_I2_ = 0.28	*K*_I2_ = 0.19
		*K*_I3_ = 0.17	*K*_I3_ = 0.10	*K*_I3_ = 0.07
		*e*_tr1_ = 0.15	*e*_tr1_ = 0.15	*e*_tr1_ = 0.15
		*e*_tr2_ = 0.30	*e*_tr2_ = 0.30	*e*_tr2_ = 0.30
PID		*K*_P_ = 0.77	*K*_P_ = 0.44	*K*_P_ = 0.35
		*K*_I_ = 0.54	*K*_I_*=* 0.207	*K*_I_ = 0.132
		*K*_D_ = 0.80	*K*_D_*=* 0.50	*K*_D_ = 0.40

**Table 2:** Parameters of water supply system controllers.

Controller	Parameters
PID/PI/I	*K*_P1_ = 0.5, *K*_I1_ = 0.135, *K*_D1_ = 0.9, *K*_P2_ = 0.3, *K*_I2_ = 0.17, *K*_I3_ = 0.05, *e*_tr1_ = 0.6 V, *e*_tr2_ = 1.2 V
PID	*K*_P_ = 0.5, *K*_I_ = 0.135, *K*_D_ = 0.9

## Data Availability

Data are contained within the article.
